# Influence of gestational weight gain and maternal depression on offspring BMI trajectory from birth to 3 years: A longitudinal mother-infant study

**DOI:** 10.1371/journal.pone.0333819

**Published:** 2025-09-30

**Authors:** Ting Zeng, Ting Huang, Liangqiong Deng, Li Xiong, Peipei Zeng, Ji Peng, Qiulan Wang, Ling Chen, Yu Zhang, Dingyuan Zeng

**Affiliations:** Department of Child Healthcare, Liuzhou Maternity and Child Healthcare Hospital, Liuzhou, Guangxi, China; Universidade de Sao Paulo Faculdade de Saude Publica, BRAZIL

## Abstract

**Objective:**

Gestational weight gain (GWG) is associated with offspring growth trajectories and early childhood obesity, but existing studies rarely account for maternal psychological status, which may confound or modify this association. This study aimed to evaluate the impact of GWG patterns on offspring body mass index-for-age Z score (BMI-Z) trajectories from birth to 3 years, adjusting for maternal depression and examining effect modification by depressive symptoms.

**Methods:**

This mother-infant paired longitudinal cohort study was conducted between September 2019 and September 2024 at the Liuzhou Maternity and Child Healthcare Hospital. Data on maternal sociodemographic characteristics, health status, lifestyle factors, clinical and anthropometric measurements were collected. Linear mixed-effects models were used to assess the association between GWG patterns and offspring BMI trajectories, adjusting for maternal pre-pregnancy BMI, sociodemographic factors, and maternal depression status. Stratified analyses by depression status were conducted to explore effect modification.

**Results:**

A total of 1,133 mother-infant pairs were included in this study. Offspring of mothers with excessive GWG showed higher BMI-Z scores after 24 months. Children born to underweight mothers had lower BMI-Z scores throughout follow-up. Mixed-effects modeling indicated that both pre-pregnancy BMI and GWG pattern were independently associated with BMI-Z trajectories (*P* < 0.05). After adjusting for depression scores, the association between overweight/obesity (β = 0.48, 95% CI: 0.23–0.76, *P* < 0.001) as well as excessive GWG (β = 0.17, 95% CI: 0.04–0.29, *P* < 0.05) and child BMI trajectory persisted. Maternal underweight was still negatively associated with child BMI z -scores (β = −0.35, 95% CI: −0.52 – −0.15, *P* < 0.001). Stratified analyses showed that the effect was more pronounced in mothers with depressive symptoms (*P* < 0.05).

**Conclusion:**

Excessive GWG significantly influences early childhood growth patterns. Maternal depressive symptoms may partially confound or modify this association. These findings highlight the need to integrate psychological assessments into prenatal care to optimize offspring growth trajectories and reduce the risk of early-life overweight.

## 1. Introduction

Early childhood growth is a critical window that influences long-term health outcomes, including obesity, metabolic syndrome, and cardiovascular risks. The prevalence of obesity in children and adolescents was reported to be 8.5% (95% confidence interval [CI]: 8.2–8.8), with a 1.5-fold increase in the prevalence of obesity from 2012 to 2023 compared to 2000–2011 [1]. Maternal factors such as pre-pregnancy body mass index (BMI), gestational weight gain (GWG) as well as mental health status have been increasingly recognized as key determinants of offspring growth [2,3]. The Institute of Medicine (IOM) provides GWG recommendations based on pre-pregnancy BMI [4], yet many pregnant women fail to meet these guidelines, either gaining insufficient or excessive weight during pregnancy [5]. It was reported that the prevalence of inadequate GWG in the USA, Europe and Asia was 21%, 18% and 31%, while excessive GWG was 51%, 51% and 37%, respectively [6]. In China, 57% of pregnant women exceed the recommended GWG, whereas 13.7% gain insufficient weight [7], leading to concerns regarding maternal and child health [8–10]. Given these high prevalence rates, understanding the long-term implications of GWG on offspring growth trajectories is of significant clinical and public health importance.

Numerous studies have demonstrated associations between maternal nutritional status and offspring growth and development, and this association has been consistently replicated across diverse populations [11–13]. It was reported that infants born to mothers with excessive GWG are more likely to have macrosomia, higher birth weight, and increased adiposity, predisposing them to obesity and metabolic disorders in childhood and adulthood [14,15]. In addition, excessive GWG alters DNA methylation and influences foetal and neonatal body composition, and maternal DNA methylation was associated with foetal total thigh and arm tissues and subcutaneous thigh and arm fat, as well as with neonatal fat mass percentage and fat mass. Moreover, excessive GWG was associated with a higher risk of childhood atopic dermatitis (odds ratio (OR) = 1.05, 95% CI: 1.01–1.10) and neurodevelopmental disorders (OR = 1.12, 95% CI: 1.06–1.19) [16,17]. Conversely, inadequate GWG is linked to preterm birth, low birth weight, failure to initiate breast-feeding, and growth restriction, and may increase offspring's intellectual developmental disorders risk, which may have long-term consequences on neurodevelopment and physical growth [8,18,19]. However, despite extensive research on biological mechanisms (e.g., placental function, fetal programming), the role of maternal psychological status—particularly depressive symptoms—remains underexplored in this context.

Emerging evidence indicates that maternal depression during pregnancy and the postpartum period is associated with altered maternal metabolic profiles, suboptimal gestational behaviors (e.g., poor diet, reduced physical activity) [20], and disrupted mother-infant interactions [21], all of which may contribute to offspring obesity risk [22–24]. Nevertheless, existing studies examining GWG-offspring BMI associations have primarily focused on sociodemographic and biomedical covariates (e.g., maternal age, parity, gestational diabetes), while neglecting the potential confounding or effect-modifying role of psychological factors [25,26]. This critical gap limits the validity of current findings and calls for a more integrative approach regarding how different GWG patterns influence postnatal growth trajectories, particularly using BMI-for-age Z-scores (BMI-Z) as standardized growth indicators.

BMI-Z is a validated index to assess child nutritional status and growth in comparison with a reference population, providing a dynamic understanding of child development across time [27]. Evaluating BMI-Z trajectories allows for identification of patterns such as rapid weight gain, plateauing, or faltering growth, which may be influenced by prenatal exposures [28]. Evidence indicates that deviations in maternal weight before and during pregnancy can alter intrauterine environments, thus modifying the child's growth potential [25]. In addition, previous studies have primarily focused on the impact of total GWG on birth outcomes, but fetal growth rates vary across trimesters, suggesting that weight gain patterns may be more informative than total GWG alone [29]. Therefore, this study aims to address two key questions: (1) Does the association between GWG patterns and offspring BMI trajectories from birth to 3 years persist after adjusting for maternal depressive symptoms? and (2) Does maternal depression modify this association, suggesting a potential effect modification? This study utilized a mother-infant paired longitudinal design with repeated measures of offspring BMI (0–3 years) and prospective assessments of maternal depression assessed via Self-Rating Depression Scale (SDS) (gestational weeks 20–28 and 32–36) and Edinburgh Postnatal Depression Scale (EPDS) (42-day postpartum). By integrating psychological data into the analysis, this study seeks to provide a more comprehensive understanding of the multidimensional determinants of early-life adiposity and inform future interventions targeting both physiological and mental health during pregnancy.

## 2. Methods

### 2.1 Study design and setting

This study was conducted in strict accordance with the ethical principles outlined in the Declaration of Helsinki. The research protocol received formal approval from the Institutional Review Board of Liuzhou Maternity and Child Healthcare Hospital (No. Fast Review-Research-2018–093). This mother-infant paired longitudinal cohort study was conducted between September 2019 and September 2024 at the Liuzhou Maternity and Child Healthcare Hospital, a tertiary obstetrics and gynecology and child health care center in Liuzhou, Guangxi, China. As a referral center for the city and surrounding rural areas, the hospital serves a region with strong socioeconomic diversity, representing the sample characteristics of the maternal and infant population in the area.

### 2.2 Participants and follow-up

Inclusion criteria were: (1) singleton pregnancy; (2) complete gestational weight records from early, mid, and late pregnancy; and (3) agreement to participate in postnatal follow-up. Exclusion criteria included: (1) pre-existing metabolic disorders (e.g., diabetes, thyroid disease); (2) preterm birth (<37 weeks of gestation); and (3) congenital anomalies affecting growth. Sample size was estimated for a repeated-measures design with 10 time points, assuming a moderate effect size (Cohen's f = 0.15), a power of 80%, and a significance level of 0.05, the minimum required sample size was approximately 900 mother-child pairs. Considering a non-response rate of 15%, the target enrollment was set to 1050.

**Fig 1:**
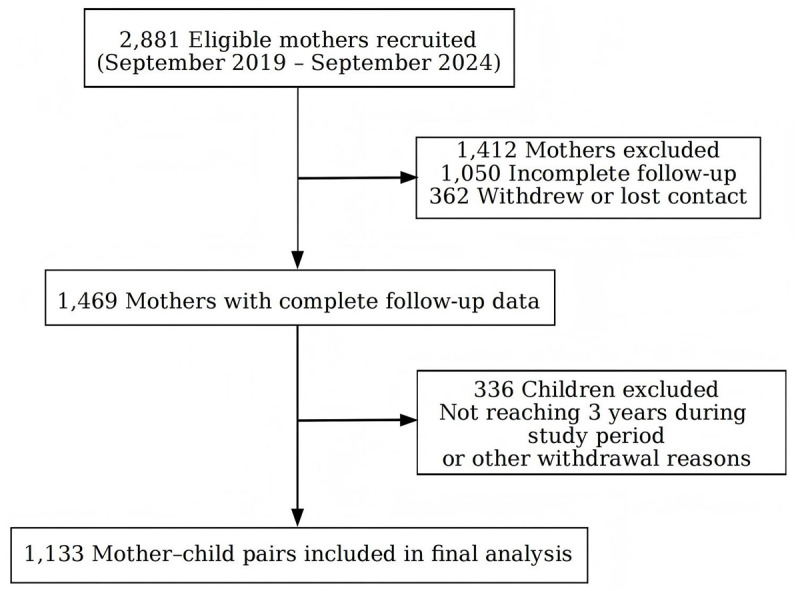
Study cohort deviation.

Eligible women were identified during routine antenatal visits by research nurses who screened medical records and conducted in-person eligibility assessments. All participants voluntarily participated in the study and provided written informed consent before enrollment. Follow-up assessments were conducted at 1, 3, 6, 8, 12, 18, 24, 30, and 36 months after delivery through scheduled clinic visits. Of 2,881 eligible mothers, 1,469 completed all follow-up visits. A total of 336 children were lost to follow-up (due to not reaching age 3 within the study period or other reasons), leaving 1,133 mother-infant pairs in the final analysis. Detailed participant flow is presented in the flowchart (Fig 1).

### 2.3 Data collection

Data on maternal sociodemographic characteristics, health status, lifestyle factors, and depression status were collected via standardized questionnaires administered during prenatal visits and postpartum follow-up. Clinical and anthropometric measurements were extracted from medical records. All measurement staff received centralized training before data collection. Anthropometric instruments were calibrated weekly. Data entry was double-checked by independent personnel, and 5% of records were randomly audited for accuracy. Internal validation was performed by re-measuring a random subset of participants, yielding intra-class correlation coefficients (ICCs) >0.95 for all anthropometric variables.

### 2.4 Exposure variables

Pre-pregnancy BMI was calculated from self-reported weight and height at the first prenatal visit and categorized per WHO standards into: Underweight: < 18.5 kg/m², Normal weight: 18.5–24.9 kg/m², Overweight/obese: ≥ 25 kg/m². GWG was calculated as the difference between the last prenatal weight and pre-pregnancy weight. Based on IOM guidelines, GWG was classified as: inadequate, adequate, and excessive, specifically, 12.5–18 kg, 11.5–16 kg, 7–11.5 kg, and 5–9 kg for women with pre-pregnancy BMI classified as underweight, normal weight, overweight, and obese (≥ 30 kg/m^2^), respectively [4].

### 2.5 Outcome variable

The main outcome was child’s BMI trajectory from birth to 3 years. BMI at each visit was calculated as weight in kilograms divided by length in square meter. Each BMI measurement was cleaned by calculating gender-and age-standardized BMI z-score using the Chinese Center for Disease Control and Prevention Anthro standards based on child weight and length at each time point.

### 2.6 Covariates

#### 2.6.1 Maternal and sociodemographic factors.

Covariates included age at pregnancy, education level (High school and below/Colleges and above), per capita monthly income < ¥5000, menstrual regularity, parity (primiparous/multiparous), planned pregnancies, methods of conception (Natural conception/Artificial conception), primiparous, regular physical activity before pregnancy (< 1 hour/ 1–2 hour/ > 2 hours), passive smoking exposure over 15 minutes, drinking during pregnancy (Never/Every once in a while), vaginal bleeding in mid-pregnancy, iron deficiency anemia in pregnancy, sleep quality at 42 days postpartum measured by Athens Insomnia Scale (AIS) [30,31], delivery mode (Natural birth/ Cesarean delivery), infant sex, and feeding type at 3 months (Exclusive breastfeeding/Mix-feeding).

#### 2.6.2 Psychological variables.

Mid-pregnancy and late-pregnancy depressive symptoms were assessed using SDS [32,33], a 20-item validated tool generating raw scores between 20 and 80 points, which can be converted into index scores ranging from 25 to 100 points, as recommended by Zung, by simply multiplying the raw score by 1.25. Depression was defined as the total index scores of SDS ≥ 50.

Postpartum depressive symptoms were measured by the EPDS [34], a 10-item tool widely used screening scale for antepartum and postpartum depression with overall score ranging from 0 to 30 [35]. Prior validation studies suggest a cutoff score of 10 or higher for possible depressive disorder.

### 2.7 Statistical analysis

All statistical analyses were conducted using IBM SPSS software (V.26.0) and R software (V.4.4.3). Descriptive statistics were used to summarize maternal baseline characteristics across prepregnancy BMI categories, reported as means ± standard deviations (SD) or medians (interquartile ranges) for continuous variables, and frequencies (percentages) for categorical variables. Differences across groups were tested using one-way ANOVA, Kruskal–Wallis tests, or chi-square tests, as appropriate. Average BMI z-scores across time were visualized by pre-pregnancy BMI and GWG group.

To analyze children’s growth patterns in relation to GWG categories—inadequate, adequate, and excessive—we employed linear mixed-effects regression models, incorporating an interaction term between the main independent variable and time (follow-up points). Each mother–infant pair was treated as a random effect to allow for individual variability in intercepts across children. The child’s BMI z-score (continuous) served as the dependent variable, while maternal BMI and GWG category (categorical) were the primary independent variables.

Linear mixed-effects models are well-suited for trajectory analyses due to their flexibility and robustness in handling longitudinal data. Specifically, this approach accounts for the correlation between repeated measurements within the same individual and accommodates variability in growth trajectories across subjects. Both child age and the GWG category or maternal BMI were treated as fixed effects, as were covariates including maternal and sociodemographic factors and maternal depressive symptoms. For interaction analyses involving maternal BMI or GWG and child age, normal-weight mothers with adequate GWG were used as the reference group. Three sequential models were constructed: Model 1 examined the association between maternal BMI and GWG category (independent variables) and the child’s BMI z-score trajectory (dependent variable), adjusting for age and sex; Model 2 extended Model 1 by additionally adjusting for sociodemographic characteristics and pregnancy history; Model 3 further adjusted for the mean SDS scores and EPDS scores to account for maternal psychological status. To explore whether depressive symptoms modified the association between GWG and child BMI trajectories, interaction terms (GWG pattern × SDS category [≥50 vs. < 50]) were tested. If a significant interaction was detected (*P* for interaction < 0.10), stratified analyses were subsequently conducted.

Sensitivity analysis was conducted and included excluding mothers with extreme GWG values (≥ ± 2 SD from the mean), and replacing continuous SDS/EPDS scores with binary variables (depression: Yes/No). Effect estimates were reported as *β* coefficients with corresponding 95% CI. A two-sided p-value < 0.05 was considered statistically significant.

## 3. Results

### 3.1 Characteristics of study population

A total of 1,133 mother-infant pairs were included in the final analysis. Based on maternal pre-pregnancy BMI, participants were divided into three groups: underweight (n = 221), normal weight (n = 759), and overweight/obese (n = 153). GWG patterns were classified as inadequate, adequate, and excessive according to the 2009 IOM guidelines, with distribution as follows: inadequate (n = 216), adequate (n = 538), and excessive (n = 379). Table 1 summarizes the baseline characteristics across BMI categories. Mothers with normal BMI in early pregnancy and with adequate GWG during pregnancy constituted the largest BMI-GWG group, n = 366 (48.2%). Mothers with pre-pregnancy overweight/obesity were more likely to have higher age, irregular menstruation, cesarean delivery, and preeclampsia (*P* < 0.05). In contrast, underweight mothers tended to be younger, and were more likely to report inadequate GWG (*P* < 0.05).

**Table 1:** Characteristics of study populations.

Variables	Total (n = 1133)	Underweight (n = 221)	Normal weight (n = 759)	Overweight (n = 153)	*P* value
Age at pregnancy (years)	30.18 (3.54)	29.35 (3.34)	30.28 (3.47)	30.88 (3.96)	<0.001
Primiparous (n, %)	720 (63.5)	152 (68.8)	478 (63.0)	90 (58.8)	0.123
Early pregnancy weight (kg)	51.81 (7.27)	43.17 (4.09)	52.04 (4.68)	62.96 (4.85)	<0.001
Height (m)	1.57 (0.09)	1.59 (0.05)	1.58 (0.05)	1.54 (0.22)	<0.001
Early pregnancy BMI (kg/m^2^)	20.82 (2.71)	17.26 (1.23)	20.92 (1.43)	25.49 (1.32)	<0.001
Total GWG (kg)	14.82 (5.55)	17.79 (8.55)	14.49 (4.18)	12.20 (3.97)	<0.001
GWG pattern (n, %)					<0.001
Inadequate	216 (19.1)	50 (22.6)	160 (21.1)	6 (3.9)	
Adequate	538 (47.5)	111 (50.2)	366 (48.2)	61 (39.9)	
Excessive	379 (33.5)	60 (27.1)	233 (30.7)	86 (56.2)	
Educational level with colleges and above (n, %)	452 (39.9)	89 (40.3)	306 (40.3)	57 (37.3)	0.773
Per capita monthly income < ¥5000 (n, %)	861 (76.0)	171 (77.4)	573 (75.5)	117 (76.5)	0.838
Exposure to secondhand smoke (n, %)	293 (25.9)	69 (31.2)	188 (24.8)	36 (23.5)	0.121
Drinking during pregnancy (n, %)	346 (30.5)	65 (29.4)	230 (30.3)	51 (33.3)	0.699
Regular physical activity (n, %)		28.79 (7.91)	29.42 (7.95)	36.80 (7.91)	0.976
< 1 hour	185 (16.3)	38 (17.2)	123 (16.2)	24 (15.7)	
1-2 hour	482 (42.5)	90 (40.7)	327 (43.1)	65 (42.5)	
> 2 hours	466 (41.1)	93 (42.1)	309 (40.7)	64 (41.8)	
Menstrual regularity (n, %)	829 (73.2)	158 (71.5)	573 (75.5)	98 (64.1)	0.012
Planned pregnancies (n, %)	686 (60.5)	118 (53.4)	468 (61.7)	100 (65.4)	0.037
Natural conception (n, %)	995 (87.8)	198 (89.6)	666 (87.7)	131 (85.6)	0.510
Vaginal bleeding in mid-pregnancy (n, %)	219 (19.3)	49 (22.2)	136 (17.9)	34 (22.2)	0.230
Preeclampsia (n, %)	282 (24.9)	54 (24.4)	178 (23.5)	50 (32.7)	0.045
Iron deficiency anemia in pregnancy (n, %)	341 (30.1)	63 (28.5)	236 (31.1)	42 (27.5)	0.567
Depression during mid-pregnancy (n, %)	370 (32.7)	65 (29.4)	248 (32.7)	57 (37.3)	0.282
Depression during late-pregnancy (n, %)	390 (34.4)	73 (33.0)	260 (34.3)	57 (37.3)	0.690
Natural birth (n, %)	746 (65.8)	163 (73.8)	495 (65.2)	88 (57.5)	0.004
Exclusive breastfeeding (n, %)	549 (48.5)	118 (53.4)	356 (46.9)	75 (49.0)	0.234
Postpartum depression (n, %)	558 (49.2)	120 (54.3)	367 (48.4)	71 (46.4)	0.224
Postpartum insomnia (n, %)	732 (64.6)	137 (62.0)	492 (64.8)	103 (67.3)	0.557
Male sex (n, %)	571 (50.4)	106 (48.0)	380 (50.1)	85 (55.6)	0.335
Birth weight (kg)	3.29 (1.08)	3.28 (1.23)	3.30 (1.09)	3.35 (0.70)	0.866
Birth length (cm)	50.72 (4.78)	50.79 (5.32)	50.77 (4.87)	50.36 (3.23)	0.608
Birth BMI (kg/m^2^)	12.56 (1.19)	12.45 (1.21)	12.57 (1.16)	12.67 (1.30)	0.175

BMI, body mass index; GWG, gestational weight gain; SDS, Self-Rating Depression Scale; EPDS, Edinburgh Postnatal Depression Scale.

### 3.2 Offspring BMI-Z trajectories across maternal BMI and GWG groups

**Fig 2:**
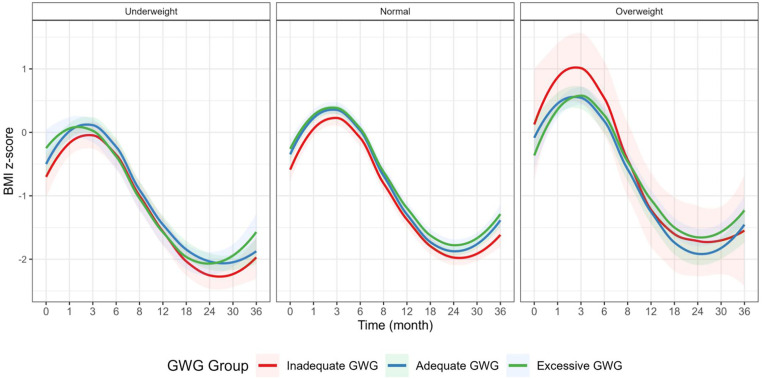
Offspring BMI-Z trajectories from birth to 36 months of age. BMI, body mass index; GWG, gestational weight gain.

Longitudinal analysis revealed distinct BMI-Z trajectories among children of different maternal BMI and GWG groups (Fig 2): Across all groups, BMI-Z increased rapidly from birth to 3 months, decreased gradually between 3–24 months, and then increased again after 24 months. Children of underweight mothers had persistently lower BMI z-scores across all time points compared to children of normal or overweight/obese mothers. Children of mothers with excessive GWG had higher BMI z-scores after 24 months, especially at 30 and 36 months, suggesting potential early onset of overweight tendencies. The most stable and moderate BMI-Z trajectory was observed in the normal pre-pregnancy BMI group with adequate GWG, suggesting this as the reference for optimal child growth. Table 2 shows the BMI z-score among different maternal BMI groups.

**Table 2:** Distribution of BMI-Z among different pre-pregnancy BMI group.

Variables	Total (n = 1133)	Underweight (n = 221)	Normal weight (n = 759)	Overweight (n = 153)	*P* value
Birth BMI_Z	−0.66 (1.10)	−0.77 (1.10)	−0.65 (1.08)	−0.53 (1.20)	0.104
BMI_Z_1m	0.63 (1.14)	0.47 (1.10)	0.62 (1.14)	0.92 (1.10)	0.001
BMI_Z_3m	0.64 (1.18)	0.33 (1.19)	0.68 (1.19)	0.90 (1.08)	<0.001
BMI_Z_6m	−0.41 (1.20)	−0.67 (1.15)	−0.39 (1.20)	−0.16 (1.26)	<0.001
BMI_Z_8m	−0.49 (1.20)	−0.77 (1.19)	−0.45 (1.19)	−0.28 (1.26)	<0.001
BMI_Z_12m	−1.36 (1.16)	−1.51 (1.22)	−1.35 (1.15)	−1.18 (1.14)	0.023
BMI_Z_18m	−1.81 (1.13)	−2.00 (1.14)	−1.77 (1.12)	−1.69 (1.14)	0.011
BMI_Z_24m	−1.96 (1.13)	−2.18 (1.18)	−1.92 (1.14)	−1.84 (0.96)	0.004
BMI_Z_30m	−1.72 (1.46)	−1.92 (1.56)	−1.70 (1.44)	−1.53 (1.39)	0.031
BMI_Z_36m	−1.51 (1.51)	−1.87 (1.44)	−1.42 (1.55)	−1.39 (1.33)	<0.001

BMI, body mass index.

### 3.3 Linear mixed models: Effects of pre-pregnancy BMI and GWG on BMI-Z

To quantify the influence of maternal pre-pregnancy BMI and GWG patterns on BMI z-scores over time, 3 models were constructed using mixed-effects linear models with BMI-Z as the dependent variable, incorporating repeated measures at all time points (shown in Table 3).

**Table 3:** Linear mixed-effect regression model of BMI Z-score.

Variables	Model 1		Model 2		Model 3	
	β (95% CI)	*P* value	β (95% CI)	*P* value	β (95% CI)	*P* value
Maternal BMI group						
Underweight	−0.32 (−0.51–-0.14)	0.001	−0.34 (−0.53–-0.16)	<0.001	−0.35 (−0.52–-0.15)	<0.001
Overweight	0.48 (0.23–0.73)	<0.001	0.49 (0.24–0.74)	<0.001	0.48 (0.23–0.76)	<0.001
GWG pattern						
Inadequate	−0.12 (−0.32–0.07)	0.218	−0.12 (−0.32–0.07)	0.315	−0.12 (−0.32–0.07)	0.367
Excessive	0.11 (0.05–0.28)	0.017	0.12 (0.04–0.28)	0.015	0.17 (0.04–0.29)	0.009
Excessive GWG × SDS category	–	–	–	–	0.15 (0.07–0.21)	0.005
Excessive GWG × EPDS category	–	–	–	–	0.12 (0.02–0.30)	0.007

BMI, body mass index; GWG, gestational weight gain; CI, confidence interval.

Model 1 adjusting for age and sex;

Model 2 extended Model 1 by additionally adjusting for sociodemographic characteristics and pregnancy history;

Model 3 further adjusted for the mean SDS scores and EPDS scores to account for maternal psychological status.

In Model 1, both maternal pre-pregnancy BMI and GWG category were significantly associated with child BMI z-score trajectory over time. Compared to the normal BMI group, children born to underweight mothers had lower BMI z-scores (β = −0.32, 95% CI: −0.51 – −0.14, *P* = 0.001). Children of overweight/obese mothers had higher BMI z-scores (β = 0.48, 95% CI: 0.23–0.73, *P* < 0.001). GWG patterns were also associated with BMI z-scores: Compared to adequate GWG, excessive GWG was linked to higher BMI z-scores (β = 0.11, 95% CI: 0.05–0.28, *P* = 0.017). However, there was no significant association between Inadequate GWG and BMI z-scores.

In Model 2, after adjusting for maternal age, education, household income, parity, and pregnancy complications, the association between pre-pregnancy underweight and lower BMI-Z remained significant (β = −0.34, 95% CI: −0.53 – −0.16, *P* < 0.001). The effect of pre-pregnancy overweight/obesity was strengthened and still statistically significant (β = 0.49, 95% CI: 0.24–0.74, *P* < 0.001). The association between excessive GWG and higher BMI z-scores remained significant (β = 0.12, 95% CI: 0.04–0.28, *P* = 0.015).

In Model 3, further adjusting for maternal depressive symptoms (EPDS scores) and mean SDS scores, the association between overweight/obesity (β = 0.48, 95% CI: 0.23–0.76, *P* < 0.001) as well as excessive GWG (β = 0.17, 95% CI: 0.04–0.29, *P* < 0.05) and child BMI trajectory persisted. Maternal underweight was still negatively associated with child BMI z -scores (β = −0.35, 95% CI: −0.52 – −0.15, *P* < 0.001). In addition, higher EPDS scores were independently associated with a modest increase in offspring BMI z-score (β = 0.05 per 5-point increase, 95% CI: 0.01–0.09, *P* = 0.021).

### 3.4 Interaction and stratified analysis

A statistically significant interaction was observed between GWG pattern and maternal depressive symptoms on child BMI z-score trajectory (*P* < 0.05, shown in Table 3). Stratified analyses showed that the effect of excessive GWG on child BMI z-scores was more pronounced in mothers with elevated SDS scores (β = 0.25, 95% CI: 0.08–0.42), compared to mothers without significant depressive symptoms (β = 0.11, 95% CI: −0.01–0.24) (shown in S1 Table).

### 3.5 Sensitivity analyses

Excluding mothers with extreme GWG values (≥ ± 2 SD from the mean) did not materially change the main findings. Similarly, results were consistent when EPDS and SDS scores were included as binary variables (depression Yes/No).

## 4. Discussion

This longitudinal study of 1133 mother–child pairs explored the associations between gestational weight gain (GWG) patterns, pre-pregnancy BMI, and early childhood BMI z-score trajectories from birth to 3 years. Unlike most existing studies, our analysis incorporated maternal psychological status—measured by the SDS and EPDS—as both covariates and potential effect modifiers. This addition represents a notable innovation, as psychological factors are often neglected in similar studies, despite their known impact on both maternal health behaviors and child development outcomes. We found that excessive GWG and higher pre-pregnancy BMI were associated with steeper increases in offspring BMI z-scores during early childhood, even after adjusting for sociodemographic and obstetric confounders. Importantly, maternal depressive symptoms significantly modified these associations: among mothers with higher SDS scores (≥50), the association between excessive GWG and accelerated BMI trajectory in children was more pronounced. This suggests that maternal mental health may exacerbate the adverse impact of suboptimal GWG on offspring adiposity outcomes.

In this study, excessive GWG was associated with higher child BMI z-scores, particularly evident after 24 months of age. This finding aligns with previous studies [36,37], which reported increased adiposity and higher BMI among children born to mothers with excessive GWG. Our findings extend this knowledge by suggesting that maternal depressive symptoms may compound these effects. Depression during or after pregnancy has been associated with unhealthy maternal behaviors, including reduced breastfeeding duration, disrupted mother–infant bonding, and increased use of formula feeding—all of which may contribute to rapid weight gain in early life [21,38,39]. Moreover, depressive symptoms may influence maternal diet quality and physical activity during pregnancy, further amplifying gestational overnutrition [40]. In addition, excessive gestational weight gain may expose the fetus to hyperglycemia, elevated insulin levels, and inflammatory cytokines, programming metabolic risk from early life [41]. Moreover, women who gain excessive weight during pregnancy may be more likely to introduce solid foods early or adopt obesogenic infant feeding practices, contributing to long-term higher BMI trajectories in their children [42,43].

This study found that maternal underweight before pregnancy was significantly associated with lower BMI z-scores in children across the first three years of life, the effect persisted even after adjusting for maternal and infant confounders, underscoring that maternal undernutrition before conception may set the trajectory for suboptimal growth early in life [44]. This is consistent with earlier literature [45,46], it is also consistent with the developmental origins of health and disease hypothesis [47], suggesting that suboptimal maternal nutritional reserves may constrain fetal and early postnatal growth potential. In addition, studies have shown that maternal thinness is associated with reduced placental nutrient transfer, fetal growth restriction, and impaired postnatal catch-up growth, potentially predisposing offspring to long-term metabolic disadvantage [48]. This may reflect compromised intrauterine nutrient delivery or alterations in placental function that program growth regulation in infancy [49]. From a mechanistic perspective, maternal underweight may lead to reduced placental nutrient transport, lower fetal fat mass accretion, and altered hypothalamic appetite regulation in the fetus, ultimately affecting postnatal growth regulation [50]. Furthermore, the postnatal feeding behaviors of underweight mothers may also differ, potentially resulting in reduced caloric intake for the infant [42].

The BMI-Z trajectories revealed a non-linear pattern across all maternal BMI groups: an initial rapid increase from birth to 3 months, a decline phase from 3 to 24 months, followed by a gradual increase after 24 months. This pattern aligns with previously reported normative BMI-Z trajectories among term infants and reflects underlying physiological processes such as the adiposity rebound and postnatal catch-up growth [36,37]. Importantly, children born to mothers in the excessive GWG group exhibited higher BMI-Z scores after 24 months, potentially indicating an increased risk for overweight in later childhood. However, among the three pre-pregnancy BMI categories, children of mothers with normal BMI demonstrated the most stable and moderate BMI-Z trajectory, highlighting the importance of achieving optimal BMI before conception.

The relationship between maternal nutritional status and offspring growth is biologically plausible. Maternal underweight may lead to reduced fat stores and lower levels of leptin and insulin-like growth factors, which are essential for placental and fetal development. Conversely, excessive GWG may expose the fetus to hyperglycemia, hyperinsulinemia, and increased inflammatory cytokines, contributing to accelerated adipogenesis. These intrauterine exposures can epigenetically influence appetite regulation, energy metabolism, and fat storage in the offspring, thereby altering postnatal growth trajectories.

However, several limitations warrant consideration. First, while BMI-Z is a widely accepted anthropometric indicator, it does not distinguish between fat mass and lean mass, and further studies using body composition data would provide more comprehensive insight. Second, potential measurement errors in self-reported pre-pregnancy weight may lead to misclassification of BMI. Third, residual confounding cannot be fully excluded, particularly with respect to postnatal environmental factors such as feeding practices and physical activity, which were not fully captured. Finally, the findings may not be generalizable beyond the studied population, given regional dietary and cultural differences.

## 5. Conclusions

Maternal pre-pregnancy BMI and gestational weight gain independently affect offspring BMI-Z score trajectories within the first three years. Importantly, maternal depressive symptoms may enhance this association, suggesting that maternal psychological well-being is a critical factor to consider in the study of early growth and obesity risk. These findings support the need for integrated perinatal care that addresses both metabolic and mental health domains to promote healthy growth trajectories in offspring.

## Supporting information

S1 Table Stratified analyses of depressive symptoms during pregnancy. (DOCX)
