# Supplementary material for: Influence of gestational weight gain and maternal depression on offspring BMI trajectory from birth to 3 years: A longitudinal mother-infant study
Source: PLoS One. 2025 Sep 30;20(9):e0333819. doi: 10.1371/journal.pone.0333819 (PMC12483201; doi:10.1371/journal.pone.0333819)
Supplement: S1 Table — (DOCX) [file pone.0333819.s001.docx]

S1 Table Stratified analyses of depressive symptoms during pregnancy

| Variables | SDS ≥50 | | | |  | SDS <50 | | | |
| --- | --- | --- | --- | --- | --- | --- | --- | --- | --- |
|  | Model 1 | | Model 2 | |  | Model 1 | | Model 2 | |
|  | β (95% CI) | *P* value | β (95% CI) | *P* value |  | β (95% CI) | *P* value | β (95% CI) | *P* value |
| Maternal BMI group |  |  |  |  |  |  |  |  |  |
| Underweight | -0.30 (-0.52–-0.16) | <0.001 | -0.31 (-0.55–-0.14) | <0.001 |  | -0.22 (-0.47–-0.10) | <0.001 | -0.25 (-0.42–-0.08) | <0.001 |
| Overweight | 0.51 (0.20–0.79) | <0.001 | 0.49 (0.24–0.78) | <0.001 |  | 0.34 (0.17–0.56) | <0.001 | 0.37 (0.21–0.61) | 0.001 |
| GWG pattern |  |  |  |  |  |  |  |  |  |
| Inadequate | -0.11 (-0.30–0.11) | 0.205 | -0.10 (-0.28–0.15) | 0.322 |  | -0.17 (-0.36–0.14) | 0.213 | -0.14 (-0.31–0.20) | 0.302 |
| Excessive | 0.25 (0.08–0.42) | 0.015 | 0.22 (0.03–0.32) | 0.021 |  | 0.11 (-0.01–0.24) | 0.065 | 0.09 (-0.07–0.16) | 0.068 |

GWG, gestational weight gain; SDS, Self-Rating Depression Scale; CI, confidence interval.

Model 1 adjusting for age and sex;

Model 2 extended Model 1 by additionally adjusting for sociodemographic characteristics and pregnancy history.
